# Computational modeling of drug separation from aqueous solutions using octanol organic solution in membranes

**DOI:** 10.1038/s41598-020-76189-w

**Published:** 2020-11-05

**Authors:** Mahboubeh Pishnamazi, Ali Taghvaie Nakhjiri, Arezoo Sodagar Taleghani, Mahdi Ghadiri, Azam Marjani, Saeed Shirazian

**Affiliations:** 1 Institute of Research and Development, Duy Tan University, Da Nang, 550000 Vietnam; 2 The Faculty of Pharmacy, Duy Tan University, Da Nang, 550000 Vietnam; 3 Department of Chemical Sciences, Bernal Institute, University of Limerick, Limerick, Ireland; 4 Department of Petroleum and Chemical Engineering, Science and Research Branch, Islamic Azad University, Tehran, Iran; 5 Department for Management of Science and Technology Development, Ton Duc Thang University, Ho Chi Minh City, Vietnam; 6 Faculty of Applied Sciences, Ton Duc Thang University, Ho Chi Minh City, Vietnam; 7 Laboratory of Computational Modeling of Drugs, South Ural State University, 76 Lenin prospekt, 454080 Chelyabinsk, Russia

**Keywords:** Engineering, Chemical engineering, Computational science, Theoretical chemistry

## Abstract

Continuous membrane separation of pharmaceuticals from an aqueous feed was studied theoretically by development of high-performance mechanistic model. The model was developed based on mass and momentum transfer to predict separation and removal of ibuprofen (IP) and its metabolite compound, i.e. 4-isobutylacetophenone (4-IBAP) from aqueous solution. The modeling study was carried out for a membrane contactor considering mass transport of solute from feed to organic solvent (octanol solution). The solute experiences different mass transfer resistances during the removal in membrane system which were all taken into account in the modeling. The model’s equations were solved using computational fluid dynamic technique, and the simulations were carried out to understand the effect of process parameters, flow pattern, and membrane properties on the removal of both solutes. The simulation results indicated that IP and 4-IBAP can be effectively removed from aqueous feed by adjusting the process parameters and flow pattern. More removal was obtained when the feed flows in the shell side of membrane system due to improving mass transfer. Also, feed flow rate was indicated to be the most affecting process parameter, and the highest solute removal was obtained at the lowest feed flow rate.

## Introduction

Presence of pharmaceuticals in freshwater and wastewater has been recognized as a challengeable issue, because these types of pollutants potentially may have adverse effects on the ecosystems as well as human health^1^. Pharmaceutical contaminants contain drug residues which can be arisen from personal care products, pharmaceutical production facilities waste, and hospital waste^2^. In this regard, many research studies have been performed for evaluating the occurrence, fate, and toxic effects of pharmaceutical compounds in drinking water and wastewater streams^3,4^. Coagulation/flocculation, filtration, and sedimentation are the conventional wastewater treatment methods that are being utilized at different scales to address this challenge. Nonetheless, these techniques are inefficient for removal of the most of pharmaceutical residues due to processing problems, and complexity of the feed solution. Indeed, high-performance processing units need to be developed and used for pharmaceutical contaminants removal from effluents to comply with environmental regulations and acts.

The concentrations of pharmaceuticals that have been detected in waste streams range between ng lit^−1^ to μg lit^−1^^5^. As an extensively utilized medicine of non-steroidal anti-inflammatory drugs (NSAIDs) to reduce pain, ibuprofen (IP) has been frequently detected in surface waters and sewage with low concentrations at micrograms per liter level^6^. The degradation process for ibuprofen removal can produce up to thirteen by-products. Amongst them, two compounds have been identified to cause harmful effects. One of these toxic metabolites is 4-isobutylacetophenone (4-IBAP) which may adversely affect the central nervous system, connective tissue cells, and red blood cells^7,8^.

Several approaches have been used for sequestration of the pollutants from water and wastewater streams including adsorption and solid-phase extraction^9–11^. However, these methods have some limitations specifically at industrial scales. The most widely used treatment method is adsorption, which is an energy-consuming process in order to regenerate the applied sorbent and can cause processing problems and pollution. On the other hand, solid-phase extraction approach allows low limits of detection, while consumes considerable time and has been found to be insufficient for application in sewage treatment plants^12^. In this sense, liquid membrane-based separation strategies can be the appropriate substitute for the traditional techniques for the elimination of pharmaceutical pollutants from water. In current years, an innovative technology has been reported to separate trace concentrations of pharmaceutical molecules from water and wastewater streams. This membrane-based technology, e.g. hollow-fiber membrane systems, possesses tremendous privileges such as high analyte capacity, low consumption of organic solvent, easy handling, low analysis cost, membrane high surface area, and independently controllable flow rates^13–19^.

Williams et al. used a hollow-fiber membrane contactor with immobilized canola oil for extraction of 4-IBAP from contaminated water^20^. With simultaneous regeneration of the immobilized solvent, 4-IBAP was extracted from the aqueous phase passing through the shell side of membrane contactor. This phenomenon made this system a green process through which 80% of 4-IBAP was removed by utilizing only 70–100 mL of canola oil. In order to extract three pharmaceutical compounds (salicylic acid, ibuprofen, and diclofenac) from the wastewater, a polypropylene hollow-fiber membrane was used by Payán et al.^21^. Detection limits were determined 20, 100, and 300 ng lit^−1^ for salicylic acid, diclofenac, and ibuprofen, respectively and this process had a very low organic solvent consumption.

In addition to experimental investigation of pharmaceutical contaminated water treatment by membrane-based approaches, a mathematical model which can assess the effects of various processing parameters on the removal efficiency is required for future applications of these separation procedures. This research study aims to understand the removal of ibuprofen and its metabolite compound (4-IBAP) from an aqueous feed by contacting with an organic solvent (octanol) in a hollow-fiber membrane contactor system. In order to achieve this objective, a mechanistic modeling along with computational fluid dynamics (CFD) simulation is employed. Besides, the influence of different parameters and the flow rate of aqueous solution on the removal efficiency of IP and 4-IBAP will be evaluated.

## Development of mathematical model

**Figure 1:**
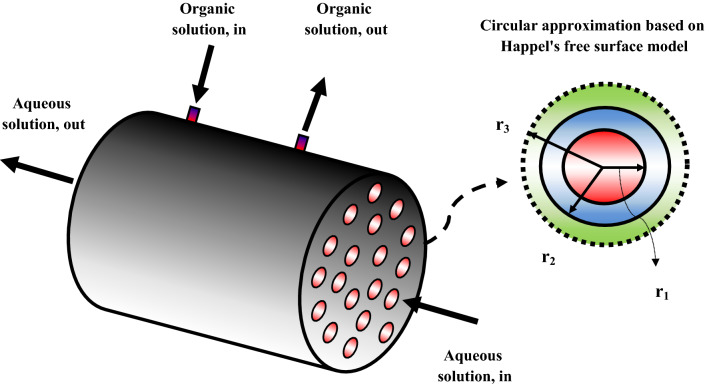
The membrane system for IP and 4-IBAP removal.

Here, the molecular removal of IP and 4-IBAP inside a membrane contactor is investigated by development of mechanistic model and CFD simulations. Figure 1 depicts the ibuprofen and 4-IBAP mass transfer and the geometrical structure of the membrane system. As seen, the tubular membrane contactor module contains many hollow fibers, however only a single fiber is considered for the modeling and numerical simulations. The aqueous feed containing pharmaceuticals flows inside the hollow fiber, whereas the octanol solution flows in the shell side, and in the opposite direction (counter current mode). However, it should be pointed out that the change of configuration (i.e. feed in the shell) will be studied using the model to evaluate the effect of flow configuration on the mass transfer efficiency. Owing to the hydrophobic nature of porous fiber, the organic phase (octanol) can penetrate into the membrane pores and reaches the other side (feed). Furthermore, as Williams et al.^8^ have reported in an experimental study, by keeping a pressure difference which is higher on the aqueous feed, the organic phase was cannot penetrate the aqueous phase and mix with the feed, as the operation must be run as non-dispersive process. As such, the extraction takes place at the interface between the organic and aqueous phases (r_1_ in Fig. 1, right side).

In this research, for estimating the effective hypothetical radius of shell encircling each membrane inside the membrane contactor, Happel's free surface model is applied^22–24^. Several simplifying assumptions have been made to develop the mechanistic model as follows:

1. Isothermal and steady state condition;
2. An axisymmetrical geometry of fiber is assumed;
3. Fluid velocity profile inside the tube side is in the fully developed condition;
4. The pores of hollow fiber are only filled with the organic phase;
5. Organic and aqueous solutions flow under a laminar regime.

The governing equation for describing IP and 4-IBAP mass transport from feed to the organic phase (octanol) is the continuity equation. This equation can be obtained as below^25,26^:

1 $$\frac{\partial {C}_{i}}{\partial t}=-{[(\nabla C}_{i}V)+(\nabla {J}_{i})]+{R}_{i}$$

where the concentration of solute, velocity vector, diffusive flux for component *i*, and term of reaction are respectively denoted as *C*_*i*_, *V*, *J*_*i*_, and *R*_*i*_. It should be noted that the term *R*_*i*_, is omitted in the model’s equations because there is no chemical reaction inside the system. In order to estimate the diffusive fluxes for component *i*, Fick’s diffusion law has been applied. Moreover, the velocity vector can be determined analytically or through combination of momentum and continuity equations. The convective and diffusional mass transports are respectively denoted by ${(\nabla C}_{i}V) \;\; \text{and} \;\; (\nabla {J}_{i})$, which have been obtained as below^25,27^:

2 $${J}_{i}=-{D}_{i}{\nabla C}_{i}$$

3 $${N}_{i}={D}_{i}{\nabla C}_{i}+{C}_{i}V$$

In the above-mentioned equation (Eq. 3), mass flux vector is defined by *N*_*i*_. The value of diffusivity can be calculated from Stokes–Einstein equation by using the solute–solvent interaction^28,29^. Based on Happel's free surface model, and assuming laminar flow regime in both shell and tube sides, velocity distribution can be derived from Navier–Stokes equation as below^25,30^:

4 $$\rho \frac{{\partial V_{z} }}{{\partial t}} - \nabla \cdot \eta \left( {\nabla V_{z} \left( {\nabla V_{z} } \right)^{T} } \right) + \rho \left( {V_{z} \cdot \nabla } \right)V_{z} + \nabla \cdot p = F\nabla \cdot V_{z} = 0$$

In Eq. (4), *V*_*z*_, *η*, $\rho$, *p*, and *F* represent the velocity vector in the *z* direction, dynamic viscosity, fluid density, pressure, and body force term, respectively. The effective hypothetical shell radius surrounding each porous fiber (*r*_*3*_) can be computed by Eq. (5) applying the Happel’s free surface model^31–33^:

5 $${r}_{3}={r}_{2}{\left(\frac{1}{1-\varphi }\right)}^{0.5}$$

In Eq. (5), $\varphi$ can be determined by Eq. (6)^31,32,34^:

6 $$1-\varphi =\frac{n{r}_{2}^{2}}{{R}^{2}}$$

where, the number of fibers and the module radius are described by *n* and $R$, respectively. The amount of *r*_*3*_ is found to be 3.158 × 10^–4^ m by combination of equations of (5) and (6).

The boundary conditions for solution of equations are listed below:

Inside fiber:

- @inlet, z = 0: $${\text{C}}_{\text{i}}={C}_{0}$$
- @outlet, z = L: $$\text{convective flux}$$
- @r = 0, axial symmetry.
- @r = r_1_, $${\text{C}}_{\text{i}}={\text{C}}_{\text{m}}/m$$

Membrane:

- @z = 0 & z = L: Insulation.
- @r = r_1_, $${\text{C}}_{\text{m}}={\text{C}}_{\text{i}}\times m$$
- @r = r_2_, $${\text{C}}_{\text{m}}= {\text{C}}_{\text{s}}$$

Shell:

- @z = L: $${\text{C}}_{\text{s}}=0$$
- @z = 0: $$\text{convective flux}$$
- @r = r_2_, $${\text{C}}_{\text{s}}= {\text{C}}_{\text{m}}$$
- @r = r_3_, $$\text{Insulation}/\text{symmetry}$$

where *C*_*s*_, *C*_*m*_, and *C*_*i*_ refer to the solute concentration in the shell, membrane, and tube side, respectively. Also, *m* refers to the partition coefficient which is defined as the ratio of concentration of solute in the organic phase to that of aqueous phase (*m* = *C*_*org*_/*C*_*Aq*_).

## Numerical simulations

The partition coefficient values of IP and 4-IBAP represented by *m*, and their values are summarized in Table 1. For solving the Navier–Stokes and continuity equations and their relevant boundary conditions in the membrane system, COMSOL package version 5.2 has been employed. The package operates based on finite element method (FEM), which is a numerical technique used for solving partial differential equations (PDEs)^35–38^. Using FEM to solve the PDEs possesses significant advantages such as easy performance of non-uniform meshes and easy handling the complicated geometries^39–41^. A 64-bit operating system with an Intel core™ i5-6200U CPU at 2.30 GHz and an 8 Gigabyte RAM was utilized for the simulations. For solution of the model’s equations, the required time has been computed to be about 1 min. Table 1 presents the physicochemical parameters and specifications of employed membrane system for developing the model and CFD-based simulations.

**Table 1:** The parameters used in the simulations^8,26^.

Parameter	Amount
Inner radius of fiber (${r}_{1}$)	11 × 10^–5^ m
Outer radius of fiber (${r}_{2}$)	15 × 10^–5^ m
Module radius ($R$)	0.0315 m
Fiber porosity	40%
Fiber tortuosity factor	2.2
Fiber length	15 cm
Number of fibers (*n*)	9950
${D}_{IP,aq}$	7.17 × 10^–10^ m^2^ s^−1^
${D}_{4-IBAP,aq}$	7.53 × 10^–10^ m^2^ s^−1^
${D}_{IP,org}$	1.47 × 10^–10^ m^2^ s^−1^
${D}_{4-IBAP,org}$	1.56 × 10^–10^ m^2^ s^−1^
${m}_{\text{IP}}$	31.62
${m}_{4-IBAP}$	37.15
${C}_{0,IP}$	10^–4^ g ml^−1^
${C}_{0,4-IBAP}$	10^–4^ g ml^−1^

**Figure 2:**
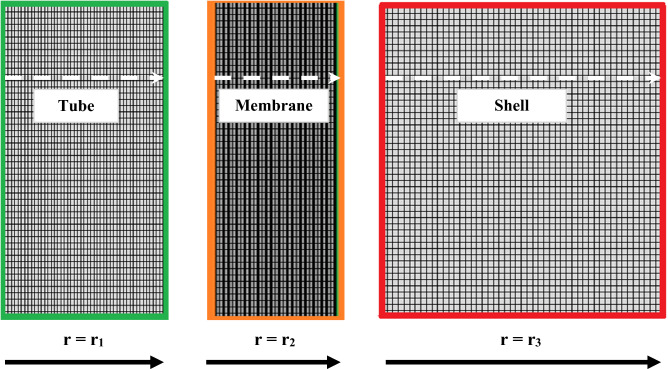
Employed mapped meshes in all domains of the contactor system.

Mapped meshing technique is aimed to be executed in this study in order to discretize the shell, tube and membrane domains of the contactor for assessing the change of important functional/design parameters at each domain point. A significant enhancement in the computational accuracy can be observed when the number of mapped meshes inside each domain of contactor increases, and thereby the calculations’ error is reduced. The meshes in three domains have been shown separately in Fig. 2.

**Figure 3:**
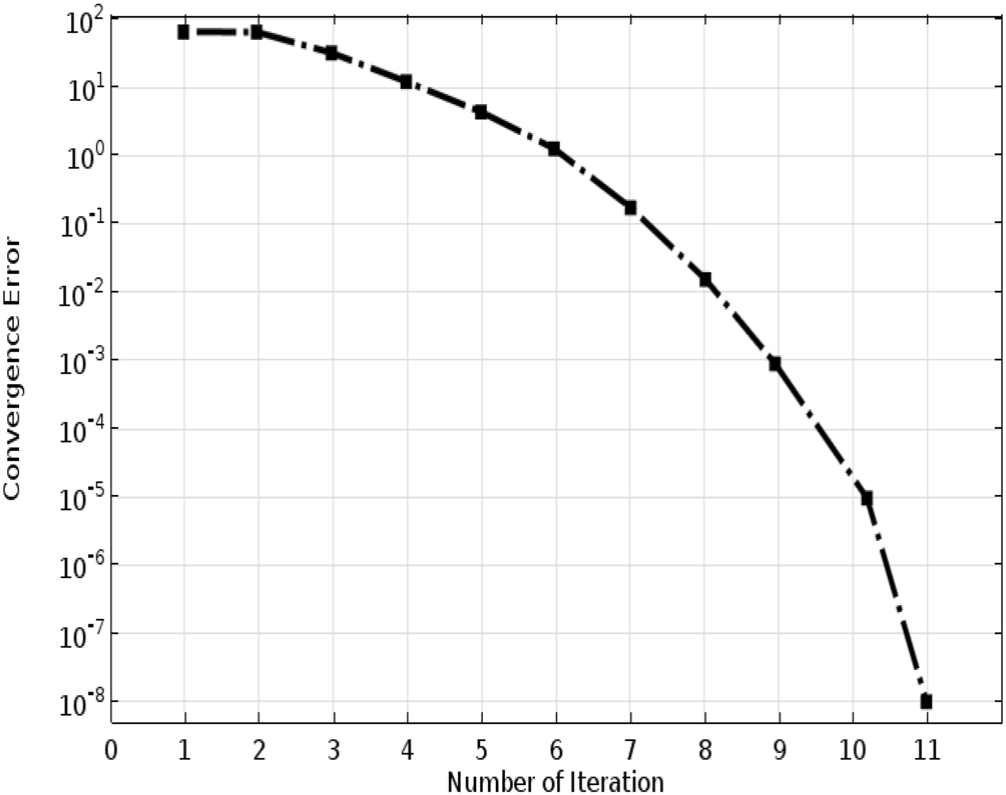
Convergence condition of CFD-based simulation.

**Figure 4:**
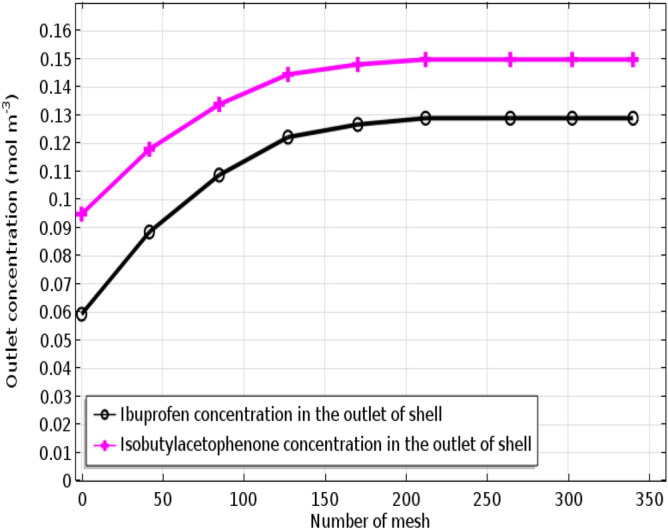
Influence of the mesh numbers on the IP/4-IBAP outlet concentration. The inlet concentrations are different.

To assess the convergence of numerical scheme used for the simulations, the convergence status of the solution as function of iteration was plotted in Fig. 3. It is seen that the system reaches the acceptable error after 11 iterations, and the solution stops at this point. This can confirm the selection of efficient solver for this set of equations developed in this study. Mesh independence test was carried out to find the optimum number of grids for the numerical solution, and also to ensure that the solution is independent of number of meshes. Indeed, increasing the number of grids in the CFD simulations would decrease computational errors and, consequently enhance the simulations accuracy. However, it would significantly increase the computational expenses. Therefore, the optimum number of meshes need to be precisely determined to avoid any computational expenses and instability. Figure 4 represents the impact of mesh numbers on the IP/4-IBAP outlet concentration. It is seen that after the 212th mesh, no meaningful variations in the outlet concentration of IP/4-IBAP concentration occurs, which corroborates the convergence of the computational simulation results. Therefore, 212 can be selected as the optimum number of meshes for the simulations, and any simulations with grids more than 212 is independent of the mesh.

## Results and discussion

### Concentration distribution of pharmaceuticals

**Figure 5:**
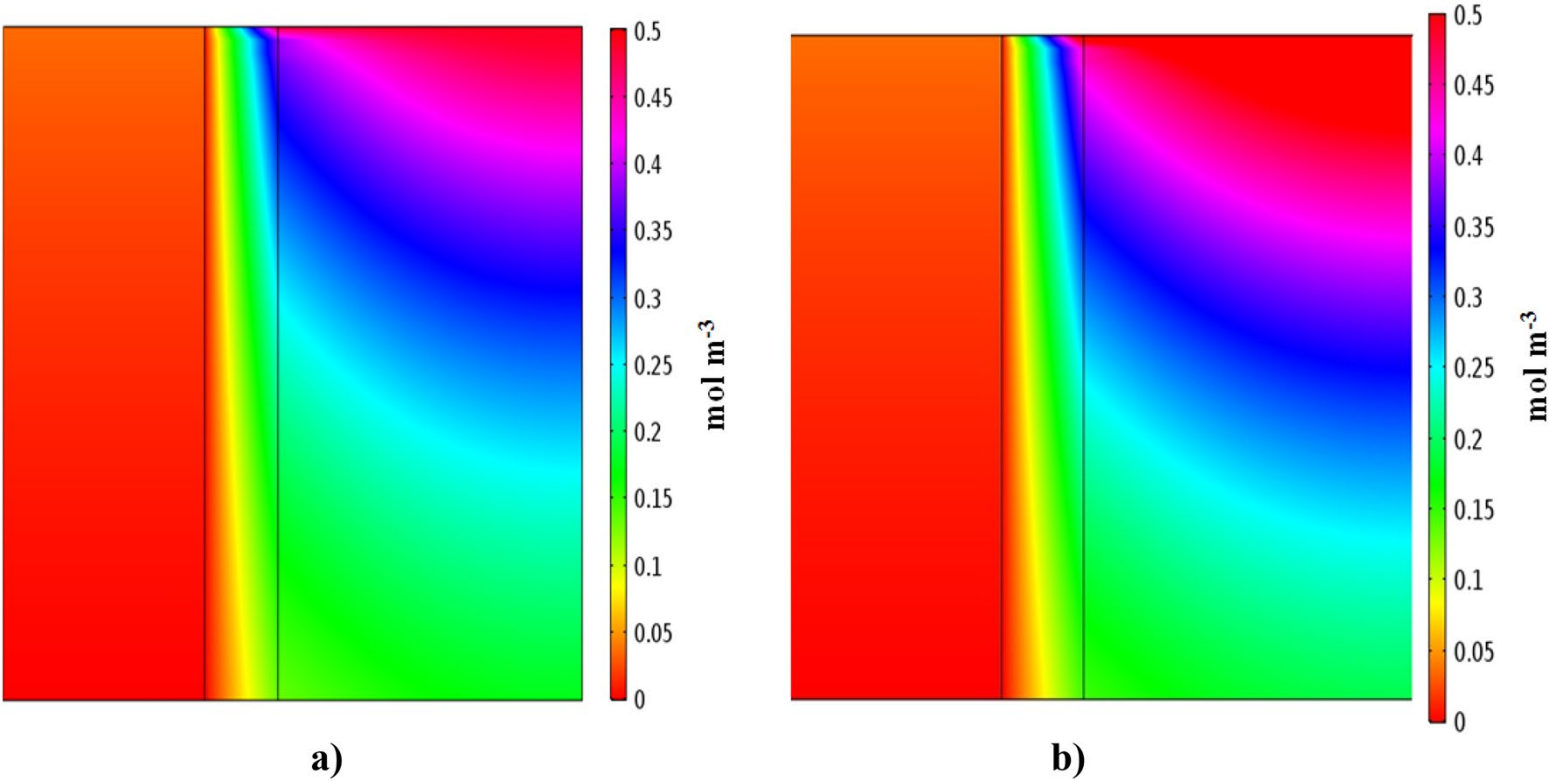
Concentration distribution of (**a**) ibuprofen and (**b**) isobutylacetophenone. Q_aq_ = 50 L min^−1^, Q_org_ = 50 L min^−1^. Aqueous solution flows in the shell side.

**Figure 6:**
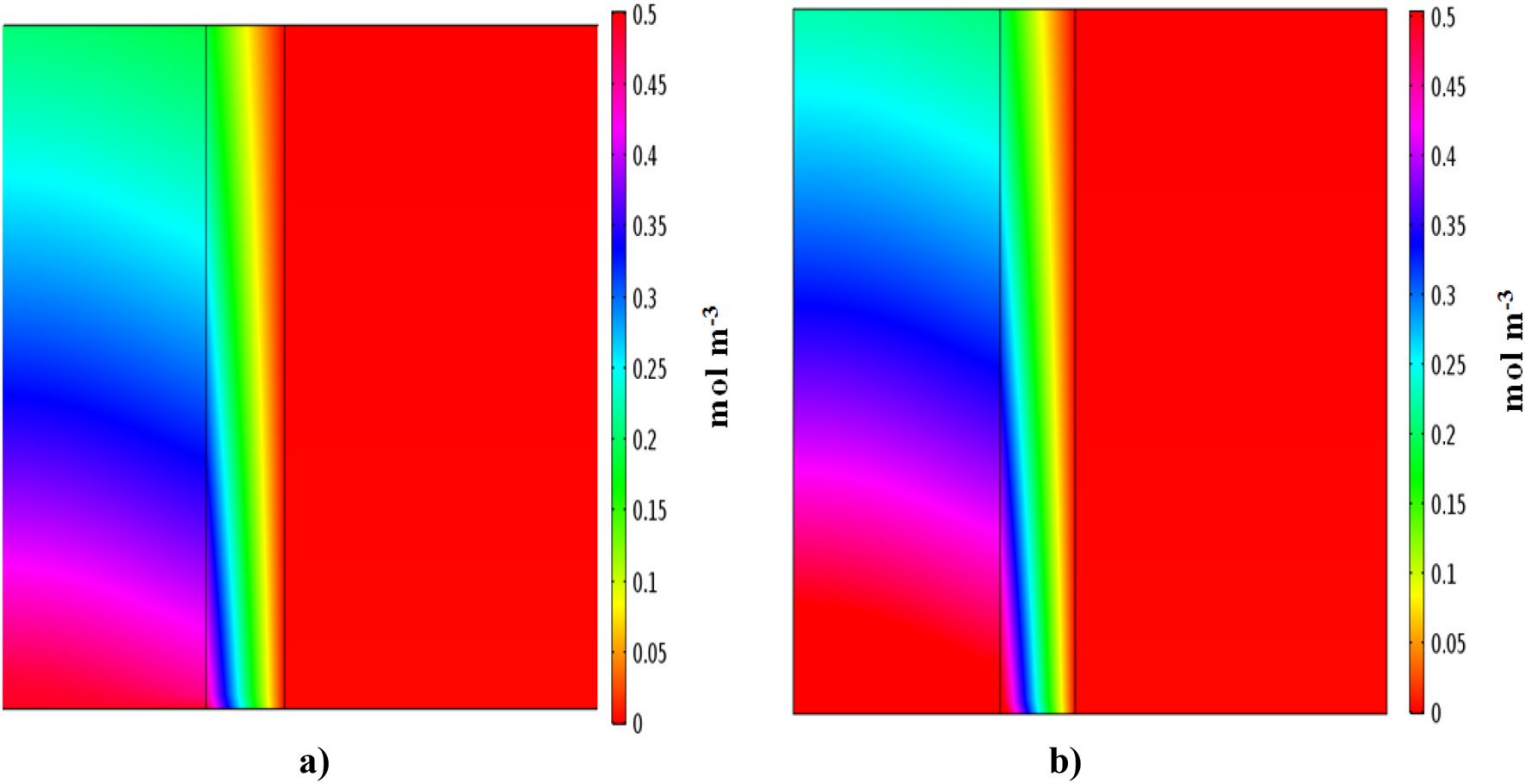
Concentration distribution of (**a**) ibuprofen and (**b**) isobutylacetophenone. Q_aq_ = 50 L min^−1^, Q_org_ = 50 L min^−1^. Aqueous solution flows in the tube side.

Figures 5 and 6 illustrate concentration distribution of IP and 4-IBAP in three distinct sections of the membrane contactor which have been obtained by solving continuity equation. It should be pointed out that the simulations have been conducted for two cases, i.e. when feed passes through the shell side, and tube side of the membrane contactor. Both simulation results are shown in Figs. 5 and 6. It is clearly observed that the concentration of both solutes change significantly as the feed flows in the membrane contactor. The concentration variations in the surface plots are due to mass transfer and movement of solute molecules towards the membrane, driven by concentration gradient. On the other side of membrane contactor, either tube or shell, the solute is taken away by the movement of organic phase. It is also seen that the solute concentration variations in the bulk of organic phase is not tangible due to formation of concentration boundary layer near the membrane wall. It can be observed that removal of solute is more when the feed flows in the shell side of membrane contactor, however the concentration profiles are needed to quantify the variations of concentration for both cases.

**Figure 7:**
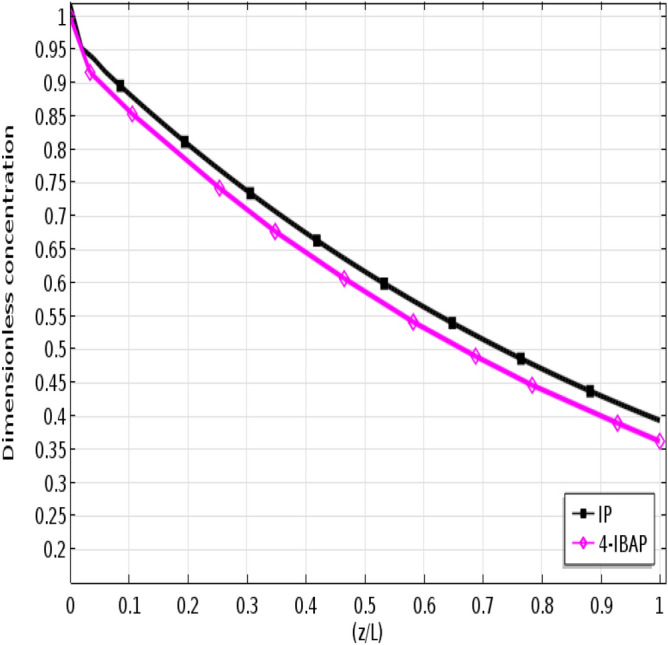
Axial dimensionless concentration profile of IP and 4-IBAP solutes along the membrane-tube interface. Q_aq_ = 50 L min^−1^, Q_org_ = 50 L min^−1^. Feed flows in the tube.

**Figure 8:**
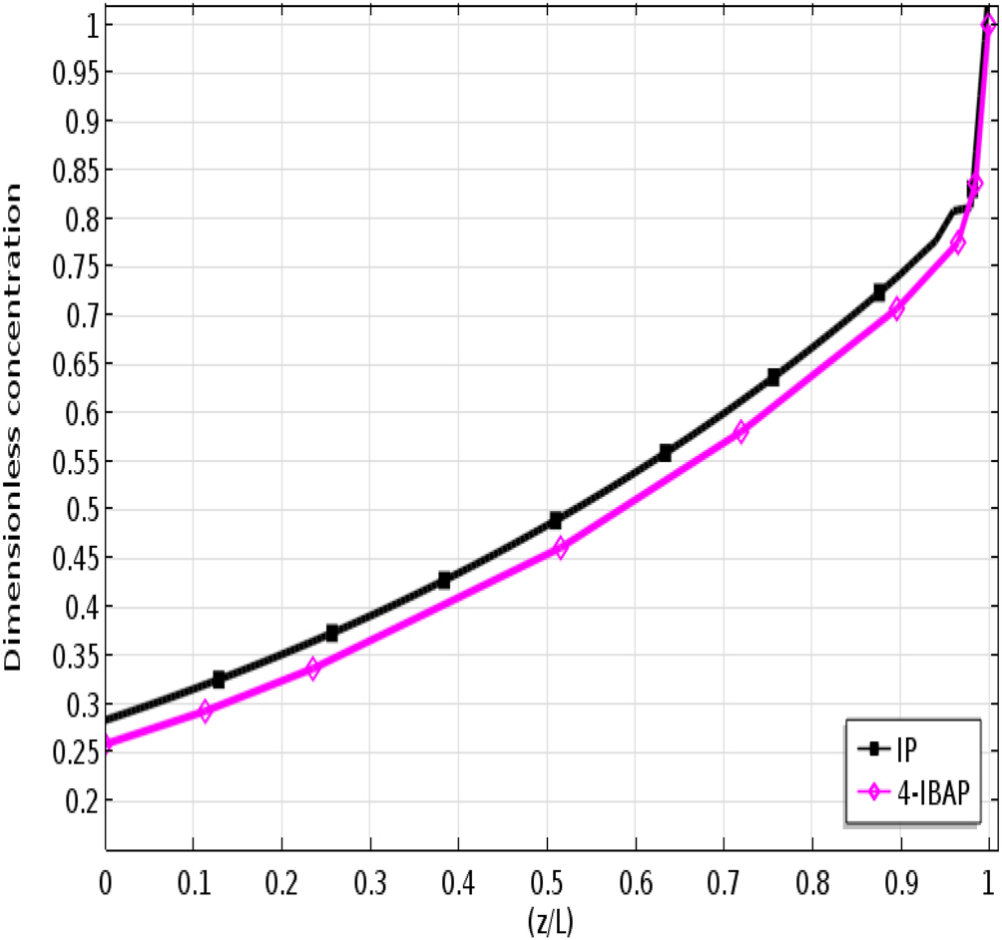
Axial dimensionless concentration profile of IP and 4-IBAP solutes along the membrane-shell interface. Q_aq_ = 50 L min^−1^, Q_org_ = 50 L min^−1^. Feed flows in the shell.

To quantify the concentration variations of each solute in the aqueous phase, axial concentration profiles are shown in Figs. 7 and 8. Figure 7 shows the concentration profile when the feed flows in the tube side, and Fig. 8 shows the concentration profile when the feed passes through the shell side. As seen, the outlet solute concentration is lower for the case when feed flows in the shell side, indicating higher removal. This could be attributed to the higher surface area for mass transfer when feed flows in the shell side. Moreover, it is seen that the removal of 4-IBAP is higher than IP which is due to higher partition coefficient of 4-IBAP in octanol.

### Effect of aqueous flow rate on separation

**Figure 9:**
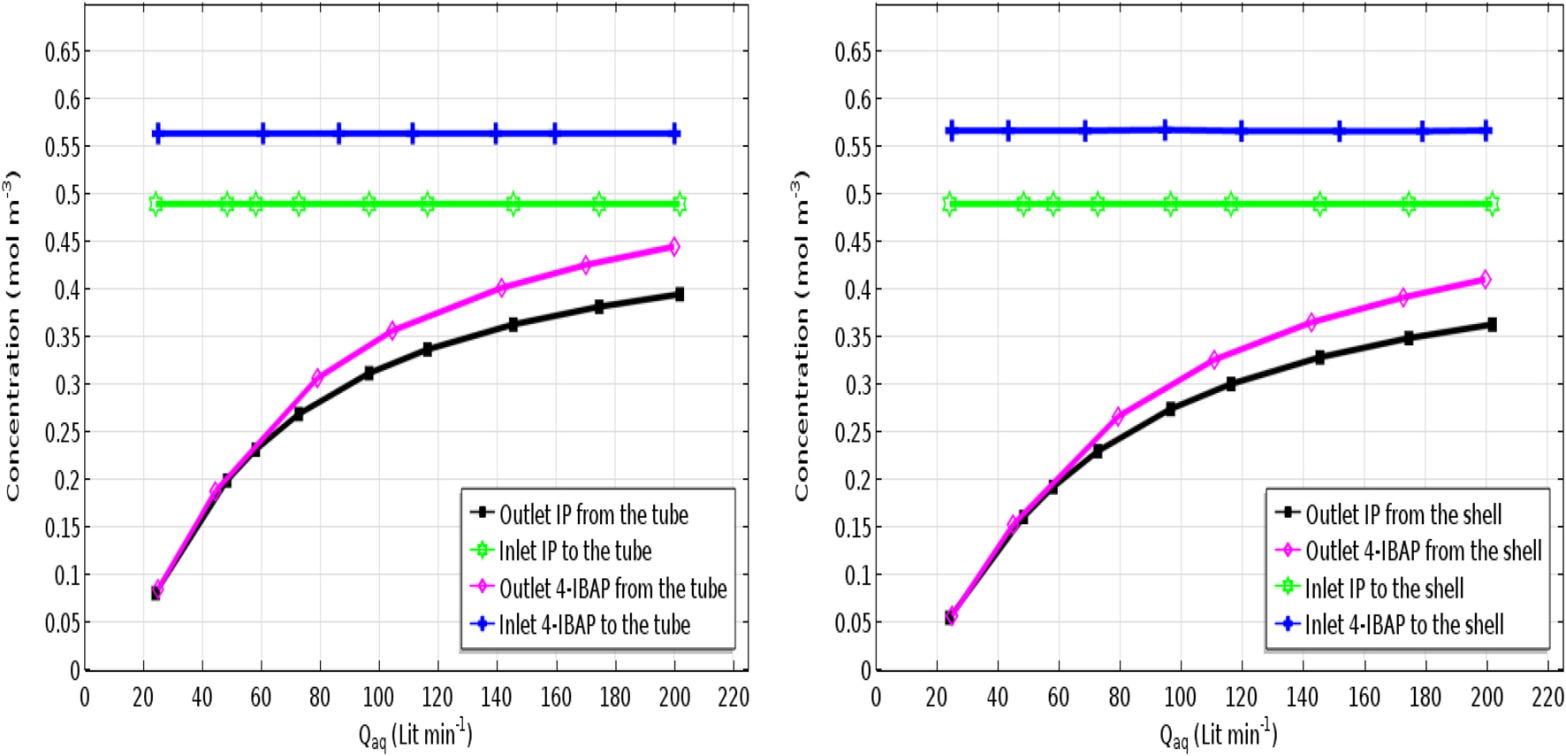
Effect of aqueous solution flow rate on ibuprofen and isobutylacetophenone outlet concentration. Q_org_ = 50 L min^−1^.

Influence of feed flow rate on the solutes removal is shown in Fig. 9 for both cases when feed flows in the tube and shell. The percentage of solute separation is determined as^42^:

7 $$Solute\;\;Separation \left(\%\right)=100\left({1-C}_{outlet}/{C}_{inlet}\right)$$

As seen in Fig. 9, the outlet concnetration of solutes is increased with increasing the feed flow rate in the membrane contactor. Indeed, separation percentages decline as the feed flow rate rises up, which is due to reduction of feed residence time in the contactor. As such, short residence time can decrease the mass transfer efficiency significantly, and in order to obtain high removal efficiency, the feed flow rate should be kept at the lowest possible value. When the residence time is long, the feed solution has more time to allow the solutes transfer to the organic phase which in turn increases the separation percentage.

### Effect of fiber porosity

**Figure 10:**
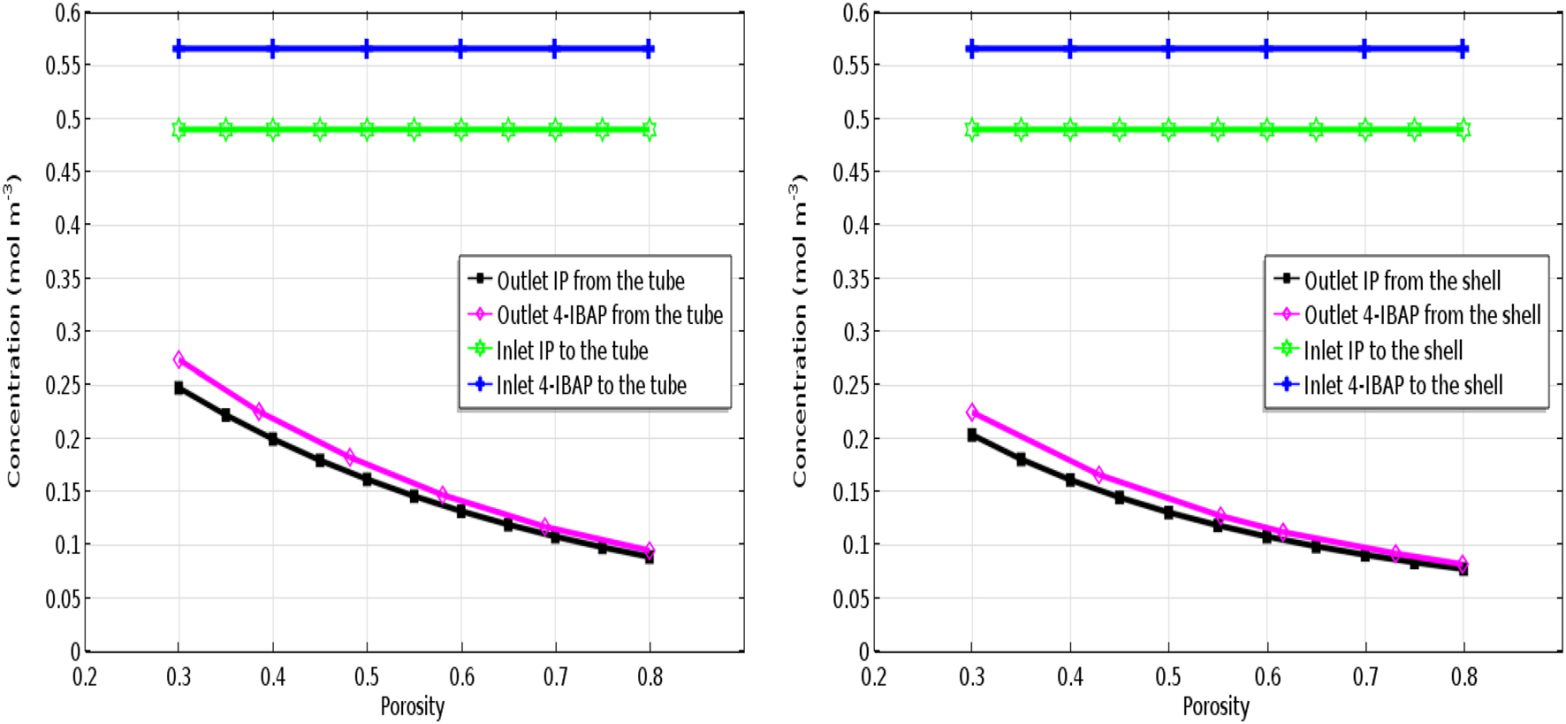
Effect of fiber porosity on the IP and 4-IBAP outlet concentration. Q_aq_ = 50 L min^−1^, Q_org_ = 50 L min^−1^.

The porosity of fiber in the membrane contactor can play crucial role in separation of solutes. It should be pointed out that in membrane contactor systems, fibers do not provide selectivity for particular components, and the role of fiber is as physical barrier for contacting two phases. However, the membrane porosity can alter the mass transfer mechanism and resistance. Using the developed model in this study, the solutes outlet concentrations at different fiber porosities were evaluated, and the results are shown in Fig. 10. As seen, the simulations for both solutes and cases have been provided. It is indicated that the porosity has positive influence on the separation efficiency of pharmaceutical components, and higher separation has been achieved at high fiber porosity values. This behavior can be attributed to the facilitation of solute mass transport by increasing porosity which decreases the mass transfer resistance. In fact, at higher fiber porosity, the collision and interaction between the solute molecules and the fiber wall would be minimized, leading to higher mass transfer rate through the pores of fiber.

### Effect of the number of fibers on IP/4-IBAP outlet concentration

**Figure 11:**
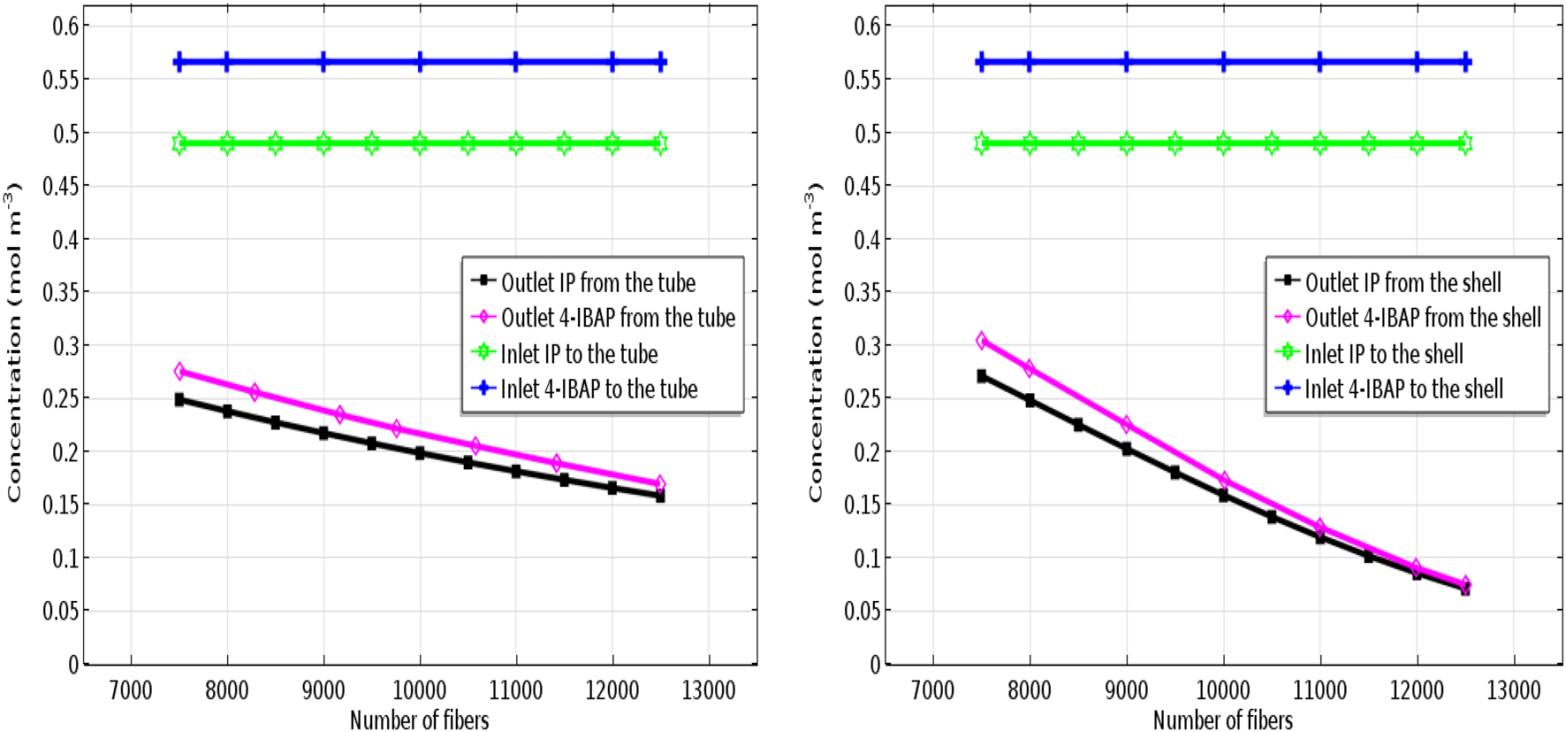
Effect of the number of fibers on IP and 4-IBAP outlet concentration. Q_aq_ = 50 L min^−1^, Q_org_ = 50 L min^−1^.

Effect of number of fibers in the contactor module, on solute outlet concentration is indicated in Fig. 11. It is shown that the outlet concentration of both solutes is decreased with increasing the number of fibers, which is favorable in mass transfer and removal of solute. In fact, variations in the number of fibers alters the mass transfer area between the feed and solvent, thereby higher mass transfer occurs with increasing the number of fibers^43,44^. It is clearly seen that for both cases, the effect of increasing the number of fibers on mass transfer is positive and can be considered as an important parameter in design and optimization of membrane-based separation of pharmaceuticals.

### Effect of the aquoues/organic flows’ arrangmement on IP/4-IBAP separation

**Figure 12:**
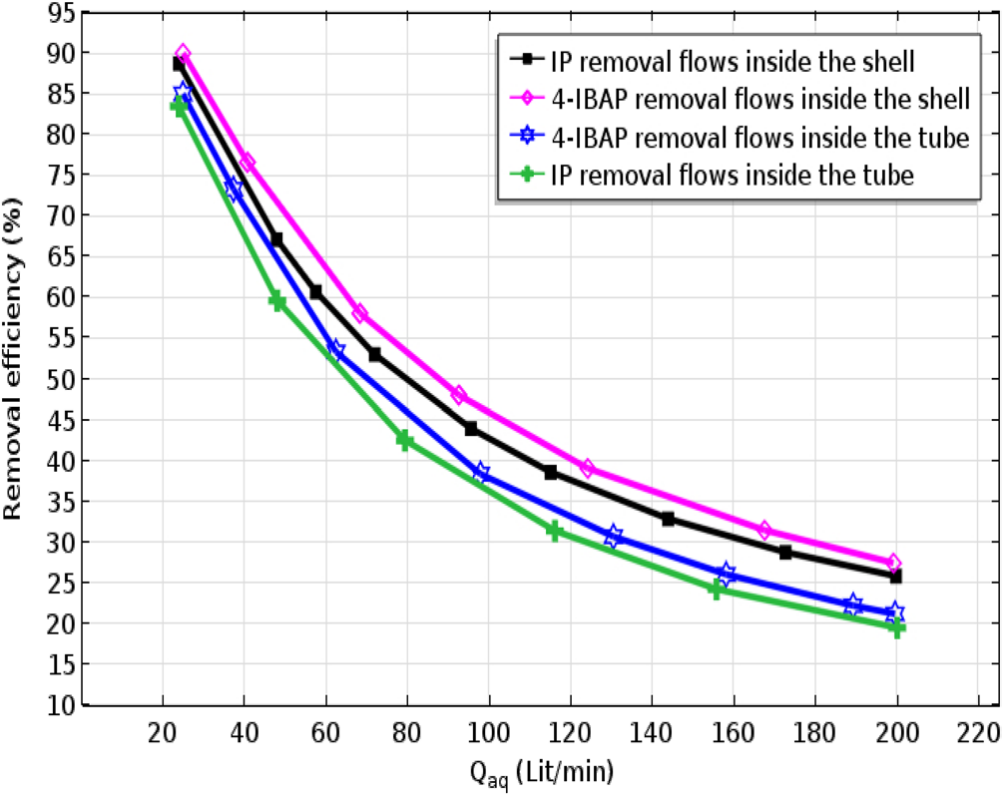
Effect of aqueous solution flow rate on ibuprofen and isobutylacetophenone removal considering different counter-current arrangements of aqueous/organic flows. Q_org_ = 50 L min^−1^.

Solutes removal efficiency as function of feed flow rate is illustrated in Fig. 12 for all operational cases. Comparisons have been made between the separation efficiency of ibuprofen and 4-IBAP when feed flows in the sell side and tube side. As indicated, the highest removal percentage has been obtained for 4-IBAP when the feed flows in the shell side. The reason for higher removal when feed flows in the shell side could be due to higher mass transfer area, and mass transfer coefficient as well. Indeed, as the membrane is hydrophobic it woud be filled with the organic phase and the interface between two phases would be formed at the fiber outer surface, when feed flows in the shell side. This will provide higher mass transfer area for removal of solutes. Furthermore, it can be seen that aqueous flow rate has major effect on the removal of solutes using the membrane systems.

## Conclusions

In this research, computational modeling drug separation in membrane contactors were carried out using computational fluid dynamics method. The mass transfer equations along with Navier–Stokes equations were derived in tubular coordinate and solved to determine the outlet concentration of solutes. Ibuprofen (IP) and 4-isobutylacetophenone (4-IBAP) which is IP’s metabolite compound were considered as solutes in the simulations. The simulations were performed for counter-current mode, but the flow pattern was changed to investigate its effect on the removal efficiency. It was revealed that higher efficiency was obtained when the aqueous feed flows in the shell side of membrane contactor which is due to higher mass transfer area. Furthermore, the simulation results indicated that lowering feed flow rate, increasing fiber porosity and number, would have positive effect on the removal efficiency of both solutes. Also, the removal of 4-IBAP was higher than IP in the membrane contactor due to its higher affinity towards the organic solvent.
